# Chronic myeloid leukemia stem cells in the era of targeted therapies: resistance, persistence and long-term dormancy

**DOI:** 10.18632/oncotarget.333

**Published:** 2011-09-22

**Authors:** Jean-Claude Chomel, Ali G. Turhan

**Affiliations:** ^1^Service d'Hématologie et Oncologie Biologique, CHU de Poitiers, France, Inserm U935, Université de Poitiers, France

**Keywords:** CML, tyrosine kinase inhibitors, leukemic stem cells, persistency, quiescence, undetectable molecular disease

## Abstract

Targeted therapies of chronic myeloid leukemia (CML) using tyrosine kinase inhibitors (TKI) have profoundly changed the natural history of the disease with a major impact on survival. Molecular monitoring with *BCR-ABL* quantification shows that a status of undetectable molecular residual disease (UMRD) is obtained in a significant minority of patients. However, it remains unclear whether these patients are definitively cured of their leukemia. Imatinib mesylate withdrawal trials have demonstrated the rapid appearance of the malignant clone in the majority of the patients whereas some patients remain in a state of UMRD. It has clearly been demonstrated that the most primitive stem cells are refractory to all TKIs used in clinical practice. In addition, long-term dormancy is one of the most fundamental characteristics of hematopoietic stem cells. In this context, we have recently undertaken a systematic analysis of the bone marrow stem cell compartment in several patients in durable UMRD. We have demonstrated the long-term persistence of a considerable amount of *BCR-ABL*-expressing stem cells, even in the absence of relapse. The phenomenon of long-term leukemic stem cell dormancy is of major importance in CML and one of the key questions in cancer biology in general. We discuss, here, the potential mechanisms, including intrinsic and microenvironmental factors, that control the response of leukemic stem cells (LSCs) to targeted therapies and potential novel strategies currently in progress with a curative intent. Moreover, we propose a molecular evaluation of the residual LSC compartment in selected patients in order to develop rational TKI-cessation strategies in CML.

## INTRODUCTION

Chronic myeloid leukemia (CML) is a major model of oncogenesis involving primitive hematopoietic stem cells (HSCs). A unique genetic alteration, the t(9;22)(q34;q11) translocation that gives rise to the Philadelphia chromosome (Ph1) generates the BCR-ABL fusion oncoprotein with deregulated tyrosine kinase (TK) activity [1]. Genetic instability phenomenon seems to be both the cause of Ph1 formation and the consequences of BCR-ABL activation [2]. Functional consequences of the t(9;22) translocation include an increased cell proliferation, a deregulation of cellular adhesion/migration and a resistance to apoptosis [3,4]. In the absence of treatment, CML classically progresses from chronic phase to blast crisis with an intermediate accelerated phase.

During the last three decades, substantial progress has been accomplished in the therapy of the disease. Allogeneic stem cell transplantation (SCT) was the first potentially curative therapy for CML patients in chronic phase [5]. However, SCT could not be applied to all CML patients because of the lack of HLA-compatible donors, but also because of immediate and late toxicities due to this process. Interferon-alpha (IFN-α), alone or associated with cytarabine, displayed real efficiency in a small number of patients, allowing durable complete cytogenetic remissions or CCyR (absence of Ph1-positive metaphase in conventional cytogenetic analysis) in patients responding to treatment [6].

Over the last ten years, small compounds such as tyrosine kinase inhibitors (TKIs) have been developed for targeted therapy of CML [7]. The introduction of imatinib mesylate (IM) has profoundly modified the natural history of CML and dramatically improved patient survival [8,9]. Nevertheless, a small percentage of patients develop primary or secondary resistance to IM therapy. Biological mechanisms of resistance are either BCR-ABL-dependent or –independent. Among them, missense mutations, identified within the ABL tyrosine kinase (ABL-TK) domain, were the most studied. More than 100 mutation affecting approximately 70 amino acids were identified [10]. The presence of these mutations modifies the protein structure and induces a conformational change of the kinase domain or disruption of the interactions between IM and BCR-ABL (affecting hydrogen or van der Waals bonds). Consequently, drug binding is impaired or totally abolished by ABL-TK mutations. Second generation TKIs such as dasatinib, nilotinib or bosutinib, were designed to overcome imatinib resistance [11-13]. These drugs are effective on most of the mutants, except for some p-loop mutations (moderate or intermediate resistance) or the gatekeeper T315I substitution. This mutation, first reported by Gorre et al. [14], was shown to confer an absolute resistance to all known ATP-competitive BCR-ABL inhibitors used in clinical practice [15,16]. More recently, a pan-BCR-ABL inhibitor TKI (ponatinib) was shown to be active against the T315I mutation [17].

In addition to mechanistic aspects of TKI-resistance due to the structure of the target oncoprotein, a novel level of complexity, inherent to the hierarchical nature of CML stem cells has been identified. This refers to the resistance of the most primitive HSCs to all TKIs currently used, because of three principal causes: their quiescence [18], the high levels of BCR-ABL expression [19] or their “oncogene-independence” [20]. Recent data showing a molecular relapse in the majority of patients upon IM-discontinuation [21] might suggest the *in vivo* persistence of leukemic stem cells (LSCs).

In the present research perspective, we focus on *BCR-ABL*-expressing hematopoietic stem cells in the context of anti-CML treatment. We analyze the question of the importance of LSCs in the maintenance of the leukemia and the development of resistance to anti-leukemia therapies. Finally, we discuss the notion of LSC persistency and the relationship between the concept of cure and the requirement of targeting leukemic stem cells.

## CHRONIC MYELOID LEUKEMIA STEM CELLS

### Elusive CML stem cell: Which cell is the real target?

CML was the first malignancy in which the concept of single primitive LSC origin was demonstrated using X-chromosome inactivation studies [22]. The finding that breakpoints between *BCR* and *ABL* genes were identical in each cell lineage of a same patient strengthened this concept. Although the involvement of T-cells has been rarely reported in CML, the occurrence of T-lymphoid blast crisis has been clearly documented [23], suggesting the involvement of a very primitive progenitor. More recently, the existence of stem cells with hemangioblast-like properties has been reported in several studies [24,25] but not confirmed by others [26]. Finally, *BCR-ABL* gene transfer experiments into both murine and human HSCs suggested that the expression of this oncogene was sufficient to initiate CML [27].

As for normal hematopoietic stem cells, CML stem cells (in the chronic phase of the disorder) have the ability to self-renew and to differentiate in committed progenitors, which produce the mature hematopoietic cells. Why and how the expression of *BCR-ABL* does not impede the mature cell production is not clear at the present time. However, the unperturbed C/EBP (CAAT/enhancer binding protein)-alpha expression in chronic phase might be involved in this event [28]. CML stem cells have been also characterized by the difficulty to examine their *in vitro* behavior as their cell surface markers are very similar to normal HSCs, which are enriched in cell populations sorted by CD34^+^CD38^−^CD71^−^, and/or Thy1^+^ markers [29]. Classically, studies involving hematopoietic progenitor and more primitive stem cell compartments have used either clonogenic (colony forming units in culture or CFU-C) or long-term culture initiating cell (LTC-IC) assays. These biological tests have shown, as expected, the presence of Ph1 chromosome in both compartments, but interestingly, they revealed the persistence of normal, Ph-1 negative stem cells at diagnosis [30] and even at later stages of the disease [31].

Although the expansion of the myeloid cell compartment occurs in the marrow, CFU-C activity is highly increased in peripheral blood at diagnosis [32]. A defect of maintenance of Ph1 clone *in vitro* as compared to normal cells was shown by LTC-IC experiments [33]. Thus, there is a clear expansion of the more differentiated progenitor and myeloid cells in peripheral blood, which certainly explains the symptoms of the disease. However, the events that lead to leukemic clonal dominance in the presence of a less amplified stem cell compartment are not established at the present time. Cell cycling studies have revealed an increased cycling of clonogenic progenitors in CML as compared to normal ones [34]. Analyses of more primitive stem cells compartments using immunodeficient mouse transplantion assays have not been very successful in CML, as they have been in acute myeloid leukemia [35,36]. These results were probably due to the difficulties of engraftment of such cells in NOD/SCID mouse, either due to cell rejection or homing deficiency of CML progenitors. Therefore, LTC-IC assays using murine stromal feeders [37] undoubtedly represent the most stringent stem cell assays in human CML.

### LSC quiescence and bone marrow microenvironment: A guilty cross-talk?

Long-term preservation of a normal hematopoiesis is based on the crucial property of HSCs to escape from the cell cycle and to remain quiescent in the bone marrow microenvironment (or niche). The quiescence phenomenon refers to a reversible cell cycle arrest (G0 phase), whereas dormancy concept represents the metabolic state of quiescent cells. Although not exactly synonymous, quiescence and dormancy are now used indifferently in human biology. One of the major characteristics of CML stem cells is the presence of highly quiescent primitive stem cells, which can be present at diagnosis in peripheral blood [38]. The majority of LSCs appears to be in cell cycle, whereas normal HSCs are generally quiescent [39]. It has been reported that bone marrow microenvironment could regulate some HSC properties such as quiescence, self-renewal, expansion, and survival [40]. This regulation takes place in the osteoblastic and vascular niches [41], which might irrespectively control highly quiescent [42] and more active HSCs [43]. Recently described nestin-expressing mesenchymal stem cells could represent a unifying concept between osteoblastic and vascular niches [44].

**Figure 1:**
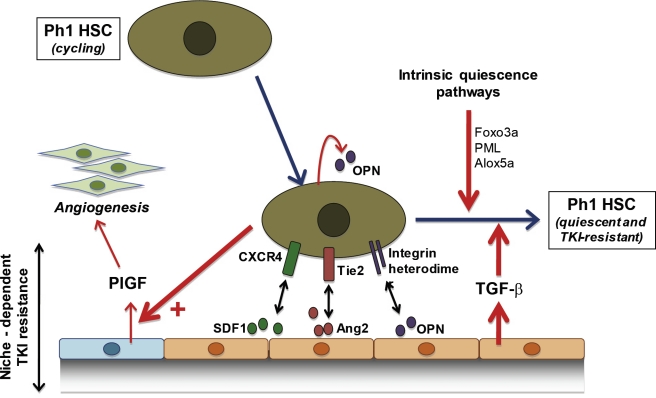
CML stem cells in the context of bone marrow microenvironmental niche Current data suggest that leukemic marrow “niche” contributes to LSC persistency via induction of a quiescence or via protective growth factors (see text). HSC, hematopoietic stem cell; PlGF, placental growth factor; CXCR4, C-X-C chemokine receptor type 4; SDF-1α, stromal cell-derived factor-1alpha; Tie2, tyrosine kinase with immunoglobulin and EGF homology domains; ang2, angiopoietin 2; OPN, osteopontin; TGF-β, Transforming growth factor-beta.

Interactions between receptors expressed by HSCs and ligands produced by marrow niches have been extensively studied [45]. The osteoblastic niche produces various soluble ligands that can act as growth factors for leukemic cells or recognize specific receptors on the surface of HSCs [46]. For example, Tie2 (tyrosine kinase with immunoglobulin and EGF homology domains), CXCR4 (C-X-C chemokine receptor type 4) and particular integrin receptors respectively binds Angiopoetin, SDF-1α (stromal cell-derived factor-1alpha) and Osteopontin produced in the bone marrow niche (Figure 1). Normal HSCs expresses the CXCR4 receptor, which recognizes SDF-1α (abundantly secreted in the marrow microenvironment), allowing the homing of HSCs to bone marrow niches. Importantly, the quiescence/cycling balance of normal hematopoietic stem cells prevents the exhaustion of the stem cell pool and critical components controlling these events are progressively being discovered. This is the case for JAM-B (junction adhesion molecule B) expressed by stromal cells, controlling quiescence [47] and Agrin expressed by marrow mesenchymal stem cells (MSCs) and endosteal cells, recognizing a specific receptor on the most primitive HSC [48].

In CML, the BCR-ABL oncoprotein decreases the adhesion of LSCs to the bone marrow environment through constitutive phosphorylation of several focal adhesion-associated molecules [49]. However, very little is known concerning the interactions between CML stem cells and the niche components. We have previously shown that *BCR-ABL*-expressing CML cells secreted high levels of osteopontin, a component of the hematopoietic niche capable to induce HSC quiescence [50] (Figure1). It has also been shown that BCR-ABL down-regulates the CXCR4 receptor in a TK-dependent manner, affecting the migration of leukemic cells towards SDF-1α *in vitro*, this effects being reversed by the use of IM [51]. In a *BCRABL*-expressing cell line model, it has been suggested that the expression of CXCR4 under the influence of IM, could generate a migration of the cells escaping apoptosis, towards bone marrow niches, this “homing” inducing quiescence and CML stem cell survival [52].

The influence of the bone marrow niche on the behavior of CML stem cells has recently been the subject of an intense debate. This microenvironment can certainly favor the survival of CML stem cells by providing survival and quiescence factors (such as TGF-β) but the notion of intrinsic abnormalities of the niche in CML has not been extensively studied. Despite the fact they are not a part of the malignant Ph1 clone, MSCs from CML patients are abnormal and secrete large amounts of PlFG (placental growth factor), which promotes marrow angiogenesis and CML proliferation [53]. Therefore, the niche might provide survival factors to CML stem cells probably in the context of LSC/niche cross-talk. Finally, some diffusible factors such as TGF-β provided by the microenvironment, could activate quiescence pathways on LSC, via nuclear translocation of forkhead O transcription factors (FOXO) [54].

## TARGETING LEUKEMIC STEM CELLS: REQUIRED FOR CURE?

Although TKI therapy (IM and 2^nd^ generation inhibitors) demonstrated great efficacy in most chronic phase CML patients, data from first line IM trials show that 15% of patients resist to the therapy [9]. Classically, the mechanisms of resistance/refractoriness are drug dependant, BCR-ABL dependent or BCR-ABL independent (Table 1) [55]. A second level of resistance is now known to occur in the very primitive stem cell level; a finding documented *in vitro* and *in vivo* as observed in IM-cessation trials.

**TABLE 1:** Mechanisms of CML resistance to imatinib

**Imatinib-dependent (decreased intracellular level)** Increased drug efflux (over-expression of ABCB1 and ABCG2 transporters) Decreased drug influx (under-expresion of OCT1 transporters) Imatinib plasma sequestration
**BCR-ABL-dependent** BCR-ABL gene amplification BCR-ABL kinase domaine missense mutations ATP binding site or p-loop (e.g. G250E, Q252E/H, Y253F/H, E255K/V.….) Imatinib binding sites (e.g. T315I, F317L,….) Activation loop (e.g. M351T, F359C/V,….) Catalytic loop (e.g. H396P/R,….)
**BCR-ABL-independent** Genetic instability Leukemic stem cell quiescence Contribution of the leukemic stem cell niche Alternative signaling pathways (e.g. independence from BCR-ABL “oncogene addiction”)

### CML stem cells are refractory to TKI therapies

*In vitro*, it has been shown that the most primitive quiescent LSCs are intrinsically refractory to imatinib [18,56], dasatinib [57], nilotinib [58] and bosutinib [59]. However, it should be noted that dasatinib retains a better efficacy in the stem cell compartment than imatinib. Multiple mechanisms can play a role in the innate resistance of primitive HSCs toward TKIs including down-regulation of OCT-1 transporter (allowing IM transport inside cells), up-regulation of ABCB1 (MDR1) and ABCG2 efflux drug transporters or high levels of BCR-ABL protein present in these cells [60,61]. In a transgenic mice model, restricting the expression of BCR-ABL in stem cell antigen 1 (Sca1)-positive cells, the authors generated a murine CML intrinsically resistant to IM [62]. Some unknown epigenetic changes induced in the context of CML cells could explain these findings and remain to be explored. In addition, a recent work has shown that the survival of Ph1-positive LSCs might be independent of the BCR-ABL kinase activity [20].

### ABL-TK mutation in CML stem cells

Early clinical observations in CML patients have suggested that ABL-TK mutations can occur in Ph1 stem cells. We have demonstrated, *in vitro*, the occurrence of ABL-TK mutations (Q252E and M351T) in CD34^+^CD38^−^ cell populations and their LTC-IC-derived progeny [63]. These results were later confirmed in CD34^+^ cells from CML patients using subcloning-PCR [64]. Consequently, it is now clear that BCR-ABL mutations arise in the primitive hematopoietic compartment. Concerning the T315I gatekeeper mutation, we have performed an extensive analysis of CFU-C and LTC-IC assays on bone marrow and blood cells from a CML patient using an allele-specific RT-qPCR (Reverse transcription-quantitative real-time PCR) [65]. We clearly demonstrated that this substitution has occurred in a very primitive Ph1 stem cell without altering its myeloid and erythroid terminal differentiation potential [31].

Overall, these data suggest that primitive quiescent CML stem cells (with or without ABL-K mutations) are protected from targeted therapies under the influence of cell-autonomous pathways or external cues such as niche-driven mechanisms, leading to the accumulation of a genuine leukemic reservoir. In addition, recent data concerning the “oncogene-independence” of these cells suggest that TKIs alone, although extremely efficient in the bulk of the disease burden, will not theoretically lead to a complete eradication of the disease. The current development of strategies targeting CML leukemic stem cells using specific pathways is based on this concept.

## TARGETING STEM CELL MOLECULAR PATHWAYS

Many signaling pathways are involved in the basic properties of HSCs, some of them could be specific of LSCs. Consequently they represent a target for potential therapy against CML stem cells [66,67].

### Some signaling pathways common to leukemic and normal HSCs

The canonical inter-connected Wnt/β-catenin, Sonic Hedgehog and Notch signaling pathways play crucial roles in the maintenance of stem cell functions. Wnt/β-catenin pathway is required for survival and long-term renewal of CML stem cells [68,69]. In the blast crisis phase of the disease, it has been suggested that hematopoietic progenitors could re-acquire some stemness properties (e.g. self-renewing) through an activation of β-catenin [70]. Recently, Hes1 (a Notch target gene) was shown to maintain CML stem cell self-renewal and promote blast crisis transformation [71]. The importance of the Sonic Hedgehog pathway has also been demonstrated in LSC renewal and CML progression. [72,73]. Moreover, the hematopoietic niche could regulate Notch and Wnt signaling pathways [42,74]. The well-known PML tumor suppressor involved in promyelocytic leukemia through the PML-RARα rearrangement has also been shown to be required for the maintenance of normal HSCs and CML stem cells [75]. Targeted therapy against normal and leukemic stem cells could now be considered since many critical signaling pathways involved in the maintenance of stemness can be inhibited (Table 2).

**TABLE 2:** targeting some critical stem cell signaling pathways

Pathway		Target	Inhibitor	Current clinical use
**Common to all hematopoietic stem cells**	Wnt/b-catenin	CK1 (casein kinase 1α)	Pyrvinium	Helminth infections
	Sonic hedgehog	SMO (smoothened)	Cyclopamine, BMS-833923	
	Notch	Notch	RO4929097, BMS-906024, PF-03084014	
	PML	PML	Arsenic trioxide	Acute promyelocytic leukemia
**Relatively specific to CML stem cells**	Alox5 (arachidonate 5-lipoxygenase)	Alox5	Zileuton	Asthma
	Hsp90	Hsp90	Geldanamycin	
	STAT5	STAT5	Pimozide	Psychotropic

### Signaling pathways specific to LSCs

Arachidonate 5-lipoxygenase (Alox5) has been shown to be indispensable for the survival, proliferation and differentiation of CML stem cells. In addition, the inhibition of Alox5 specifically impairs LSC functions and does not affect normal HSCs [76]. The AHI-1 (abelson helper integration site 1) oncogene might also be a potential LSC target since it appears over-expressed in CML stem cells and enhance BCR-ABL effect on cell growth [77]. *In vitro*, the suppression of AHI-1 over-expression was related to a reduction of LSC growth autonomy. Moreover, AHI-1 was found to interact not only with BCR-ABL but also with the JAK2/STAT5 pathway. In another field, it has been shown that the inhibition of the Hsp90 protein (a chaperone for BCR-ABL) could target CML stem cell [78]. Finally, recent publications highlight the benefit of targeting STAT5 (signal transducers and activators of transcription 5) since this pathway has been clearly involved in leukemia cell survival and TKI resistance [79,80]. However, to be used as therapy, the effects of STAT5 inhibitors on the LSC compartment have to be verified [81].

## DYNAMICS OF THE RESPONSE TO TYROSINE KINASE INHIBITORS

The hematological follow-up of CML patients on TKI treatment is classically based on blood cell counts (estimation of the hematologic response), cytogenetic analysis by the determination of the percentage of Ph1-positive metaphases (evaluation of the cytogenetic response) and quantification of *BCR-ABL* mRNA transcripts (assessment of the molecular response). International recommendations have determined the appropriate periods of testing in order to ascertain the status of the response (optimal and suboptimal responses, failure, warnings) [82]. Moreover, a screening of ABL-TK mutations is mandatory in case of TKI failure (primary resistance) or loss of therapy response (secondary resistance) [83].

### Monitoring of treatment response

RT-qPCR is the most sensitive test for the quantification of *BCR-ABL* mRNA transcripts. Laboratory protocols are now properly standardized [84-86] and international reference controls have been elaborated [87]. A *BCR-ABL/“control gene”* ratio is determined with usually *ABL* as the control gene. The main objective of these collaborative studies was to establish a concordant major molecular response (MMR) value corresponding to a *BCR-ABL/ABL* ratio of 0.1%. Moreover, for TKI good responders, RT-qPCR remains the ultimate test to follow the residual disease. It must be underlined that the limit of detection of *BCR-ABL* quantification protocols is around 10^−5^ (*BCR-ABL/ABL* ratio ~ 0.001%). The regular quantification of the *BCR-ABL* mRNA transcript level, evaluated by a *BCR-ABL/ABL* ratio every 3 months allows the determination of kinetics of CML response throughout the disease monitoring.

**Figure 2:**
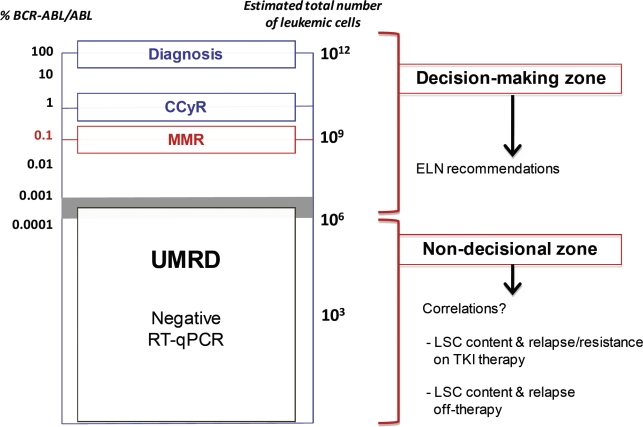
Several levels of residual disease in CML (adapted from Goldman, 2007, Blood, 110, 2828-37) On the same scale are presented together the *BCR-ABL/ABL* ratios and the estimated number of leukemic cells at diagnosis. The time of diagnosis, complete cytogenetic response (CCyR) and major molecular response (MMR) are shown on the graph. The “decision making' zone is the context of clinical decisions currently taken using ELN recommendations in CML. The “non-decisional” zone correspond currently to the clinical situation of LSC persistency in which the knowledge of the status of LSC persistence could, in the future, be of help to make clinical decisions.

A schematic representation of decreasing CML residual disease has been proposed [88]. It integrates the *BCR-ABL/ABL* ratios, the estimated total-body leukemic cell burden, the CCyR, and the MMR (Figure 2). The absence of detectable *BCR-ABL* mRNA transcript from blood and/or marrow samples using RNA samples of adequate quality characterizes an undetectable molecular residual disease or UMRD (also named complete molecular response). It is noticeable that the non-detection of *BCR-ABL* mRNA transcript does not mean the complete eradication of leukemic cells since up to one million CML hematopoietic cells could persist in the patient's body in the UMRD context.

### Mathematical models of treatment monitoring

These models are based on the kinetics of *BCRABL* mRNA transcripts determined during molecular monitoring of CML patients. For most patients responding to imatinib, a biphasic *BCR-ABL/ABL* exponential decrease is typically observed. Mathematical modeling of such observations related to the hematopoietic differentiation process (from hematopoietic stem cells to mature cells) has been elaborated [89,90]. The two slopes of the *BCRABL* mRNA decline curves (3-4 log reduction) could be consistent with the eradication of differentiated mature CML cells (fast slope) and leukemic progenitors (slow slope). Therefore, residual leukemic cells in the UMRD context are likely to correspond to CML stem cells. Using the same model, it was recently suggested that sustained TKI therapy might reduce (in part and in some cases) the leukemic stem cell burden [91]. In addition, these models propose some hypotheses concerning the relationship between leukemic progenitors and blast crisis [92], the eradication of CML stem cells [91,93], and the importance of stem cell quiescence on the success of TKI therapy [94]. However, data concerning the stem cell compartment have to be verified on biological experiments.

## LEUKEMIC STEM CELL PERSISTENCY AND CURE OF CML

Data from patients treated with allogeneic SCT, which is considered as the only “curative” therapy of CML, show that late relapses can occur up to 15 years after transplants. An unbalanced relationship between LSCs and graft versus leukemia effect, which might be operational in a fraction of LSC, could be the cause of late relapses [95]. Concerning IFN-α or TKIs (mainly imatinib), several treatment discontinuation trials have been performed. Although data from IM discontinuation trials suggest that some patients are disease-free and off-therapy for approximately 3-4 years, a significant percentage of patients relapsed (molecular or cytogenetic relapses) after the cessation of the therapy. Overall, these data point to a likely role of CML stem cells in the relapse occurrence. Therefore, investigation of the stem cell compartment in these patients seems to be warranted, in order to determine 1) the persistence of these cells in the UMRD context and 2) their relevance with regard to the occurrence of the relapse. This approach appears crucial to design rational algorithms of TKI-therapy discontinuation trials [96].

### Therapy discontinuation trials

For IFN-α therapy, clinical experiences have shown that a minority of CML patients achieved a long-term period of CCyR. In this population, it has been reported that some patients who stopped their treatment maintained a durable CCyR [97]. In this work, one patient who was in major cytogenetic response (<35% Ph1 chromosomes) at the time of cessation maintained his remission status after more than 7 years after stopping IFN-α. Although very probable, the presence of *BCR-ABL*-expressing LSC was not evaluated in these early studies. It is important to note that, before the TKI era, IFN-α therapy has induced in a few patients a prolonged CCyR that have persisted after the discontinuation of the treatment [98]. Thus, some LSC could be efficiently controlled in a “dormant” or “inactive” state in some patients treated with IFN-α alone (but not in UMRD state), suggesting a particular role for IFN-α in the therapy of CML. It has been reported that IFN-α could be more active than imatinib against leukemic progenitors [99]. Furthermore, *in vivo* mouse models are suggestive of its potential to activate quiescent hematopoietic stem cells [100]. A two-step strategy combining the activation of dormant LSCs (by IFN-α) and the destruction of re-cycling cells (by IM) has been suggested [101].

More recently, several clinical pilot studies of imatinib cessation for patients who achieved sustained UMRD have been published [102-107]. Overall, these trials showed that UMRD persists in less than half of patients. Consequently, a cytogenetic or molecular relapse was observed in the remaining. Moreover, the great majority of the patients had previously treated with IFN-α. The French STIM (STop IMatinib) trial recently reported the results of imatinib cessation in patients with sustained UMRD for 2 years or more [21]. In this work, the molecular relapse-free survival was 41% at 1 year showing that a molecular relapse occurred in a significant proportion of patients. In this study, no difference was observed according to the use or not of IFN-α as first-line therapy. These clinical trials could not only support the *in vivo* existence of LSCs believed to be responsible for the molecular relapse, but also address the issue of whether the patients who did not relapse are definitely cured or not.

### Persistence of leukemic stem cells

UMRD or complete molecular remission have been defined as non-detection of *BCR-ABL* mRNA transcript in the peripheral blood by RT-qPCR and/or nested RT-PCR [82]. This assumption is mainly related to the sensitivity of the method and the characteristics of the biological sample. Detection at the genomic level of the *BCRABL* rearrangement in some patients in UMRD after IM therapy or SCT demonstrated the presence of residual leukemic cells not detected by RT-PCR experiments [108,109]. These data show that leukemic cells are not entirely eradicated by TKIs. It must be noted that, in these experiments, *BCR-ABL* positive DNA was detected in peripheral blood. In addition, the stem cell compartment was not analyzed. Moreover, RT-qPCR experiments on purified sorted cells from CML patients treated with imatinib showed the elimination of CML progenitors within one year but the retention of the LSC population [110].

Ten years ago, (based on the observation of patients in sustained CCyR following IFN-α or allogeneic SCT), we postulated that the *BCR-ABL* rearrangement could persist unexpressed in dormant cells [111]. The persistence of such dormant leukemic progenitors in CCyR induced after IFN-α therapy had previously been demonstrated using RT-PCR experiments on CFU-Cs, [112]. More recent studies in CML patients who achieved CCyR following TKI therapy (mainly imatinib, but also dasatinib or bosutinib) have also demonstrated the persistence of Ph1 stem cells [113-115]. These experiments were performed by fluorescent *in situ* hybridization (FISH) on CD34^+^ fraction [113,114], CD34^+^ CD38^−^ cells [115] or CFU-Cs and LTC-IC [113] populations. In these studies, the presence of persistent LSCs was somewhat expected as there was no evidence of molecular eradication of the leukemia.

The UMRD status can now be obtained in CML patients treated with TKI and, have also been observed in historical cohorts, in a minority of patients treated with IFN-α. The question of whether *BCR-ABL*-expressing LSCs persist in these populations has been unexplored so far. We have analyzed the presence of *BCR-ABL*-expressing hematopoietic stem cells in bone marrow samples from six CML patients with undetectable *BCR-ABL* transcript in their peripheral blood for more than 3 years [96]. The experimental design was based on CFU-C and LTC-IC assays according to a common protocol. Long-term cultures were carried out either on classical murine MS-5 feeder cells or on HOXB4-expressing MS-5 stromal cells [37,116]. Approximately 20 individual colonies and up to 20 pools of 10 colonies were plucked from clonogenic assays at day 0 and from LTC-IC-derived progenitors at week 5. Individual and pooled colonies were tested for the presence of *BCR-ABL* mRNA by RT-qPCR and controlled by nested RT-PCR. Using this strategy, we detected a variable percentage of *BCR-ABL*-expressing stem cells in all patients analyzed. Interestingly, these primitive HSCs did not produce any detectable *BCR-ABL* transcript in the peripheral blood, suggestive of their quiescent status *in vivo*, even in patients in whom the anti-leukemic therapy had been stopped for > 11 years. It is noticeable that a greater percentage of *BCR-ABL*-expressing stem cells was observed in patients previously treated by IFN-α alone as compared with those treated by IM after IFN-α failure. These data could be indicative of a more effective eradication of the stem cell compartment in the latter case, but they must be confirmed on a larger series of patients. Finally, the major involvement of marrow LSCs in these patients (2000 CFU-Cs or LTC-ICs analyzed and only a fraction of the marrow cells assayed) suggest the presence of a highly active inhibitory process in the bone marrow niches, maintaining these stem cells in a long-term dormancy state.

## CONCLUSIONS

TKI therapy has dramatically improved the overall survival in CML and a minority of patients achieved a sustained state of UMRD. In this context, for clinicians, patients and socio-economical reasons, the recurrent question remains the discontinuation of TKI therapies. Clinical data show that half of the patients will develop a molecular relapse within a few months after imatinib cessation. Despite the fact that the decision of IM-discontinuation was done without the assessment of the LSC content in these patients, the leukemic stem cells persistency with active stem cells is highly probable in these patients. However, in the remaining 40% of patients who did not relapse, the evaluation of the LSC persistency might be of major interest. Consequently, in the context of molecular remission, the characterization of LSCs by functional assays could be considered in clinical practice. Overall, these data raise some critical questions.

### 1 - Predictive role of stem cell tests following targeted therapies

**Figure 3:**
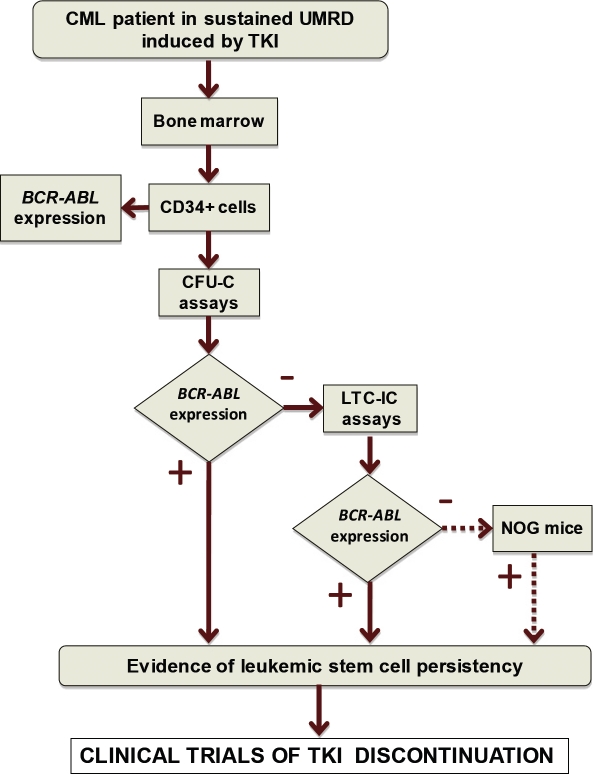
Experimental strategy to analyze the presence of *BCR-ABL*-expressing cells in progenitor and stem cell compartments UMRD, undetectable molecular residual disease; CFU-C, colony forming unit-cell; LTC-IC, long-term culture-initiating cell; NOG, NOD/Shi-*scid*/IL-2Rγ^null^.

We have developed a screening procedure based on the detection of *BCR-ABL* mRNA transcripts in CFU-Cs and LTC-ICs by RT-qPCR that appears more adapted than FISH to analyze large amount of hematopoietic colonies. To ensure the reliability of the test, RT-qPCR experiments have to be carefully controlled at each step of the amplification process (presence of positive and negative controls). However, the method can underestimate the number of *BCR-ABL*-expressing stem cells because of to the small number of hematopoietic cells analyzed (500-4000, according to the size of the colonies) and a variable transcription of *BCR-ABL* in Ph1 hematopoietic progenitors [117]. To be more efficient, we recently modified the procedure to convert it in a step-by-step protocol (Figure 3). All LTC-IC assays are now performed on MS-5/HOXB4 stromal feeders in order to increase the sensitivity of the test. In addition, LTC-IC experiments are only carried out when the preliminary CFU-C assays are negative (absence of *BCR-ABL*-expressing progenitors). In the presence of negative CFU-C and LTC-IC assays, *in vivo* xenogeneic transplantations in immunodeficient NOG (NOD/Shi-*scid*/IL-2Rγ^null^) mice can be considered. In the light of clinical data and stem cell tests, it remains to be established whether the persistency of residual *BCRABL*-expressing stem cells (cycling or quiescent) in CML patients in sustained UMRD represents or not a potential risk of disease recurrence.

### 2 - Can TKIs alone cure CML patients?

Before discussing this issue, we have to precise the notion of cure in CML. We can distinguish the “clinical cure” defined by the absence of relapse from the “biological cure” defined by the absence of leukemic cells (including LSCs). This assertion is supported by our data showing that some patients who appeared clinically “cured” had persistent LSCs. The detection of *BCR-ABL*-expressing clonogenic and LTC-IC-derived progenitors in patients with sustained UMRD might confirm the presence of a significant quiescent CML stem cell compartment. Moreover, it has been shown that the most primitive LSCs are intrinsically refractory to all TKIs used in clinical practice. In addition, TKIs might cause an osteogenic niche homing of LSCs increasing the quiescent CML stem cell burden. In this context, the use of IFN-α (to awake dormant LSCs) could be of interest. Conversely, some studies hypothesize that long-term TKI therapy (especially with the 2^nd^ generation drugs) could significantly reduce the malignant stem cell compartment. It has also been reported that it was not necessary to kill the last leukemic cell [118]. Nevertheless, TKI therapy alone seems unlikely to be fully curative, and a risk of relapse subsists even in sustained UMRD. Consequently, in this context, the molecular residual disease has to be thoroughly monitored. In this situation, positive and negative RT-qPCR areas can be distinguished (Figure 2). The first one corresponds to a “decision-making” zone and is related to ELN (European leukemia Net) recommendations. The second is consistent with a non-decisional zone in which the correlation between the leukemic stem cell persistency and relapse (on- or off-TKI therapy) remains to be evaluated.

### 3 - Leukemic stem cells as a target

Theoretically, to eradicate a leukemic process, it is mandatory to eliminate the last leukemic stem cell; this might be the definitive objective in CML [119]. Nowadays, many signaling pathways involved in hematopoietic stem cell maintenance have been described, and a few inhibitors (some in clinical use) have been identified. It is then possible to affect more or less specifically LSCs. The option of targeting CML stem cells in patients in sustained UMRD must be considered with precaution because of the probable side effects of the drugs. In this circumstance, a prior evaluation of the LSC compartment could be useful.

Despite the great success of TKI therapies, many points have to be clarified such as the mechanisms of long-term leukemic stem cell dormancy, the role of an immunomodulatory effect of the treatments, the relation between CML stem cells and the bone marrow microenvironment. Moreover, the analysis of *BCR-ABL*-expressing stem cells in CML patients with sustained UMRD induced by 2^nd^ generation TKIs must be performed, as these drugs generate molecular responses more rapidly than imatinib.
